# The Normal and Pathologic Physiology of the Hypophysis

**Published:** 1912-02

**Authors:** Joseph L. Miller

**Affiliations:** Chicago


### BUFFALO MEDICAL JOURNAL

VOL. LXVII. FEBRUARY, 1912. No. 7

### ORIGINAL COMMUNICATIONS

### The Normal and Pathologic Physiology of the Hypophysis.

JOSEPH L. MILLER, M. D.
Chicago.

THE hypophysis or pituitary body, first assumed clinical significance when Marie ascribed acromegaly to changes in this organ, although earlier writers had noted its enlargement in pathologic giants. Changes in the hypophysis are no doubt responsible for many of the giants mentioned in history, and it has played a role both in mythology and art. The sculptures on certain French and Italian churches show that the giants pictured there are in reality acromegalic individuals. This anomalous development, the giant, is in 40 per cent. of the cases, according to Sternberg, due to changes in the hypophysis.

Following the disturbances in growth, the next phase of this subject to attract attention was the discovery by Howell and Schafer of a substance in the posterior lobe capable of producing a marked rise in blood pressure and resembling in many respects extracts obtained from the suprarenal gland. These discoveries did not, however, by any means exhaust the physiologic possibilities of this gland, as recent investigations have greatly enlarged the field of its functional activity. It might be well, before discussing this organ, to refer briefly to its gross and microscopic anatomy.

Embedded in the sella turcica, the hypophysis is composed of two lobes entirely different in origin and structure. Embryologically the large anterior lobe arises from an invagination of the buccal ectoderm; the posterior lobe from an outgrowth on the floor of the third ventricle of the brain, these two portions coming in contact form the hypophysis. The anterior lobe is, therefore, an epithelial structure, arising from mouth epithelium, the posterior lobe nervous tissue. The epithelial cells are differentiated into two types, glandular and simple columnar. The anterior lobe is composed almost entirely of glandular epithelium, many of its cells containing granules characteristic of secreting cells, adjacent to the cleft there is a thin coating of columnar epithelium which is reflected onto the adjacent posterior lobe, and in the ox constitutes about one-third of its entire thickness. This columnar epithelium is known as the pars intermedia; its cells are less distinctly granular and are arranged in man in the form of islets, many of these hollow and filled with colloid. This colloid material is thought to be discharged into the infundibulum, which enters the pars nervosa, and from here passes through its canal to the third ventricle. In the cat, the infundibulum extends as a hollow canal through the pars nervosa to the pars intermedia. In the cleft between the two lobes, we not infrequently find quite large and rather firm colloid masses evidently secreted by the pars intermedia. The pars nervosa is solely nervous tissue and evidently does not have any secretory functions. In man the line of demarcation between the pars intermedia and pars nervosa of the posterior lobe is not well defined, in the ox, however, the two structures are distinct. From the above description, it is seen that we have in the hypophysis three distinct structures, the glandular anterior lobe, the pars nervosa and the intervening pars intermedia, all to be taken into consideration when dealing with the functions of the organ.

In the following short report it is impossible and inadvisable to take up a full argumentative discourse on all points under discussion, but we will rather endeavor to confine ourselves to justifiable conclusions.

First to be considered is the EFFECT OF ENTIRE REMOVAL OF THE HYPOPHYSIS. There has been considerable discussion in the past as to whether the hypophysis was an organ essential to life. The conflicting reports upon this phase of the subject can be largely accounted for, first by the difficulties of the operation with the attendant infection leading to a fatal termination, and second, the uncertainty of a complete removal. This last point is of especial importance as it has been shown that even a very small fragment of the anterior lobe will undergo rapid hypertrophy and may function sufficiently to greatly modify the final results. Only those reports can be safely accepted where after apparent complete removal and survival of the animal, a later microscopic examination fails to reveal any remnant of the gland. Even in the total removals, fragments of the pars intermedia always remain behind attached to the stalk. Present evidence indicates that when the removal is complete there follows a train of symptoms which invariably terminate fatally. The animal recovers from the immediate effect of the operation and in the case of puppies may live two or three weeks. Sooner or later, however, there is evidence of disturbance. The animal becomes apathetic, the temperature decidedly subnormal, the gait stiff and unsteady. When standing or walking the back is arched, the hind feet placed forward and walking becomes more and more difficult. The pulse is frequent and irregular. Infrequent coarse muscular twitchings may follow slight external stimuli. Finally the animal sinks into coma and very gradually dissolution occurs. It has not been possible to prevent this fatal termination by grafting or feeding hypophysis.

The posterior lobe alone may be removed without serious disturbance. The animal recovers completely and remains apparently normal. Removal of the anterior lobe, however, is followed by the same group of symptoms as total extirpation. It is evident, therefore, that the anterior lobe of the hypophysis is the portion of the organ essential to life. This might perhaps be anticipated from its glandular nature.

Certain interesting phenomena may be observed after partial removal of the anterior lobe. When the proper amount is left, the animal may continue to live for an indefinite period of time. For a few weeks after the removal, the only observed pathologic disturbance is marked polyuria without glycosuria. Later the thyroid hypertrophies, the animal gains in weight, becomes fat and apathetic, fails to come into heat and at autopsy the ovaries are atrophied, the uterus infantile and the mammae undeveloped. In such an animal, examination of the hypophyseal region shows that the pars nervosa had undergone atrophy, being replaced by hyperplastic pars intermedia. It is quite difficult to interpret the changes described. It is not improbable that the polyuria is due to hyperplasia of the pars intermedia, the gain in weight as we will see later, from atrophy of the posterior lobe. The atrophy of the reproductive organs will be discussed in detail later.

Summarizing the effect of operative procedure on the hypophysis, it can be said complete removal is followed by characteristic disturbances and fatal termination. Removal of the posterior lobe is not inconsistent with apparently normal health. Removal of the entire anterior lobe gives the same results as removal of the entire hypophysis. Removal of a portion of the anterior lobe is followed by polyuria and atrophy of the sexual organs.

### THE HYPOPHYSIS AND GLYCOSURIA.

Marie in his report on acromegaly called attention to the frequency of associated glycosuria. It is generally accepted at the present time that patients with acromegaly frequently have a glycosuria, and Borchardt in a recent review of the subject, reports that 40 per cent. of these patients have either a spontaneous glycosuria or a lessened sugar tolerance. The glycosuria in these cases, on account of its transient character is frequently overlooked, in many cases, it permanently disappears, and the amount of sugar in the urine is not infrequently independent of the character of the diet. With the presence in acromegaly of an enlarged anterior lobe, the question arises whether the glycosuria is due to pressure on the hypothetic sugar center in the region of the tuber cinereum or the result of disturbed secretion of the hypophysis. If the latter view is accepted, is the glycosuria due to the direct action of the secretion, or secondary to changes in other ductless glands? Changes in the thyroid and suprarenal are not infrequently associated with acromegaly, and both of these organs are concerned in sugar metabolism.

Although it is too early to make any absolutely definite statements on this subject, it is generally conceded that glycosuria does not accompany hypophyseal tumors except when associated with symptoms of acromegaly. As acromegaly is without much doubt due to hyperplasia and supersecretion of the anterior lobe, we should expect if the glycosuria were due to the direct action of the secretion, that extracts of this portion of the hypophysis, when administered to animals, would disturb sugar metabolism. Considerable work has already been carried out on this phase of the subject. The results however, are so at variance that it is advisable to review the matter critically. Borchardt injected subcutaneously into rabbits extracts prepared from the entire hypophysis and obtained in about 80 per cent. of the animals a more or less marked glycosuria. The sugar appeared three or four hours after the injection and rarely persisted for more than twenty hours. The extreme susceptibility of the rabbit to disturbances of sugar metabolism, modifies the significance of these results. Spontaneous glycosuria is common in rabbits, and very slight disturbance or manipulation may excite it. Lewis, Matthews and myself working on this subject found that glycosuria could be so readily excited in the rabbit that the marked disturbance following the injection might easily cause it. Borchardt later repeated the experiments on dogs. Five of the seven animals reacted with a glycosuria, but on analysis in none of these were the results actually conclusive. Two gave sugar only when on an excessive carbohyrate diet. A portion of the pancreas had previously been removed from two of the dogs, the animals, however, being sugar free. When on a proteid diet, but, after injecting extracts of the hypophysis, glycosuria appeared. Finally the other positive case, had following the injection, a very severe and prolonged tremor—a very common experience after injection of extracts from the hypophysis. This prolonged tremor by shaking out the glycogen from the muscle could readily give rise to a transitory glycosuria. Turning to other investigators, Pal and Franchini both reported negative results. With these conflicting reports Lewis, Matthews and I decided to modify these experiments by injecting separately anterior and posterior lobe in order to determine what portion of the hypophysis was responsible for the glycosuria. Dogs were injected either intraperitoneally or intravenously with freshly prepared extracts. In all, thirty animals were thus treated, fifteen with extract of the anterior lobe and fifteen with extract of the posterior lobe. In only three of these animals were we able to detect a glycosuria although a reducing substance was frequently found in the urine. The amount of sugar present as determined by the polariscope and fermentative test did not exceed ½ per cent. As none of the positive cases had a polyuria and as the glycosuria was of only a few hours' duration, the total amount of sugar eliminated was very small. Two of the positive cases received extracts of the anterior lobe; one of the posterior lobe. If we are justified in concluding from these results that extracts of the hypophysis may excite a glycosuria, it must be due to a substance present in both lobes. As the only structure common to both is the pars intermedia it may be suspected that this is the portion of the organ responsible for the glycosuria.

Turning to another phase of the subject, Goetsch, Cushing and Jackson found disturbance of sugar tolerance after removal of all or part of the hypophysis. Following the operation there was a temporary glycosuria which they attribute to setting free an excessive amount of secretion in the manipulation of the organ. On account of the readiness with which anaesthesia excites a glycosuria in animals, this must be considered as a possible factor. We have done considerable work upon this subject and find quite constantly a glycosuria in dogs after ether anaesthesia prolonged beyond an hour. They also report that after removal of the posterior lobe following the temporary glycosuria, there is an increased sugar tolerance, so that larger quantities of sugar than usual can be administered without the appearance of sugar in the urine.

Summarizing the role of the hypophysis in glycosuria, it may be said that acromegaly is very frequently associated with a glycosuria. Extracts of either the anterior or posterior lobe, when injected in dogs may occasionally cause a glycosuria. Removal of the posterior lobe apparently increases the animals' sugar tolerance.

### EFFECTS OF THE HYPOPHYSIS ON THE METABOLISM AND GROWTH OF INDIVIDUALS.

The hypophysis is no doubt an important factor in the physical development of both man and the lower animals. As has been already stated, about 40 per cent. of the so-called giants owe their abnormal development to changes in this organ. The disturbance of development may be divided into two types not entirely distinct, but rather overlapping in many results. The striking feature in acromegaly is the overgrowth, especially of the extremities. These changes are in part due to enlargement of the bones, but also in a measure to increase in the subcutaneous tissue, and in some of the individuals there is a marked tendency to general adiposity. In addition, while the external genitalia may be hypertrophied, the uterus, ovaries and testes are frequently hypoplastic. The disturbance in the hypophysis responsible for the bone changes, when they occur early in life, leads to gigantism; when taking place in adult life, to acromegaly. The other type of disturbance known as Fröhlich's syndrome, is characterized especially by adiposity and failure of development of all the sexual organs, but without bone changes, or at least without bony enlargement.

Several theories have been advanced to explain acromegaly, but at the present time it is generally conceded that the hypophysis is the cause of the disturbance. The point at issue is as to whether it is a sub- or super-secretion. On account of the presence of a tumor in the hypophysis, Marie assumed that the disturbances of growth were due to lessened secretion. It has been determined, however, that only a certain type of tumor in the hypophysis is associated with acromegaly. Quite uniformly there is an enlargement of the anterior lobe due to adenomatous development with increase in the specific glandular secretory cells. Malignant or degenerative processes do not cause acromegaly. In some cases of the disease where there is no apparent enlargement of the anterior lobe, microscopic examination may still show an excess of specific secretory cells, or the possibility of hyperplasia of the pharyngeal hypophysis, as described by Haberfeld must be considered. The effect on growth of castration or pregnancy during which time the functioning activity of the ovary is in abeyance, supports the theory of supersecretion, as in both of these conditions the anterior lobe of the hypophysis undergoes hyperplasia. This phase of the subject will be taken up more in detail later. While it has not been definitely proved, the concensus of opinion at the present time is that acromegaly is due to supersecretion of the anterior lobe.

The other disturbance of development characterized by adiposity and sexual atrophy, and known as Fröhlich's syndrome, is especially associated with new growths at the base of the brain. An animated discussion is in progress as to whether the changes mentioned are due to disturbances of the hypophysis or merely phenomena arising from pressure action at the base of the brain. A review of the literature favors implication of the posterior lobe, as in the majority of recorded cases there is evidence of lessened function of this lobe either by the direct invasion of new growths and degenerative processes, or by pressure from adjacent structures. As has been mentioned, sexual impotence and adiposity are infrequent in acromegaly and could be accounted for by pressure from the enlarged anterior lobe on the adjacent posterior lobe. Tumors in the vicinity could exert direct pressure, as has been suggested by Müller. Tumors of the cerebellum by causing an internal hydrocephalus can compress the hypophysis by forcing downward the floor of the third ventricle. It has also been shown, especially by Cushing and his colleagues, that removal of the posterior lobe in dogs is followed by genital atrophy and adiposity, the latter due according to Cushing, to resulting increased sugar tolerance.

In relation to the role of the hypophysis in modifying growth, recent studies in metabolism are of considerable interest. These investigations have been confined to acromegaly and to animals fed or injected with hypophysis. It is important in studies of this character that the animal receive the separate lobes rather than the entire hypophysis, but unfortunately in the past, the experiments have been carried out with the entire hypophysis, or at least statements are not made to the contrary. Most of this work has been done on animals for short periods of time. There are no recorded experiments where comparative studies have been made upon the effect in development of animals by prolonged feeding of the various anatomic components of the hypophysis.

It is the consensus of opinion that in acromegaly there is retention of phosphorus, calcium and magnesium, and often of nitrogen and chlorine. In some instances the retention of phosphorus and calcium is very decided. This is in accord with what we might expect from the over development in this condition.

Unfortunately we have very few feeding experiments where young animals have been given daily for a long period of time preparations of either the anterior or posterior lobe. It is not improbable that such feeding experiments might give very valuable information in regard to the effects on growth. The only experiments along this line that I have been able to find in the literature are those of Schafer. He fed to young rats for about three months dried anterior lobe, and to the controls the same amount of dried testes, and found that the former group gained considerably more in weight than the latter. We are at present conducting some feeding experiments with both the anterior and posterior lobe, which we hope may throw some further light on this subject.

Summarizing the effect of the hypophysis on growth, it may be said that supersecretion of the anterior lobe is probably responsible for the clinical picture known as acromegaly. Lessened secretion of the posterior lobe leads to adiposity and atrophy or failure of development of the sexual organs. On account of the early appearance of sexual impotence in acromegaly, it is possible that the anterior lobe may play a role in sexual activity, although this may well be explained by pressure in the posterior lobe, from the enlarged anterior lobe.

EFFECTS ON BLOOD PRESSURE OF EXTRACTS FROM THE HYPOPHYSIS.

One of the earliest discoveries in regard to the hypophysis was the effects of its extracts on blood pressure. Howell, Schafer and others demonstrated the presence of a pressor substance closely related to adrenalin. This may readily be extracted by macerating the gland with normal salt, and when injected intravenously produces a marked rise in blood pressure, more prolonged than adrenalin, due chiefly to its effects on the peripheral vessels. The heart is usually slowed, and in the majority of instances the amplitude of contraction is lessened, so that the action upon the heart is depressive in character; in this respect, it differs from adrenalin. Although extracts of the suprarenal gland, and the hypophysis both cause a rise in blood pressure; their method of action is quite different. Suprarenal extract acts upon the nerve endings in the vessels and may be inhibited by apocodein or ergotoxin; hypophysis extract effects the muscle directly, and is not inhibited by either of the above mentioned drugs. Both slow the heart, suprarenal extract through its action on the vagus, hypophysis extract by a direct depressing action. It is, furthermore, interesting to note, that while a subcutaneous injection of suprarenal extract will almost invariably relieve an attack of bronchial asthma, the hypophysis is without effect. The intensity of the peripheral constriction varies somewhat in different groups of vessels, those of the kidney undergoing dilatation, differing again in this respect from the suprarenal extract. As a result of this local dilatation in the kidney, extract of hypophysis has a marked diuretic effect especially when given intravenously. When administered by mouth, to man, its action is very uncertain and even intramuscularly, it shows an inconstant diuretic action. Schafer believes its diuretic action is independent of its effect on blood pressure, and due to the direct action of some substance on the renal epithelium. With our present knowledge of the method of action of diuretics, this seems very improbable. Polyuria, of hypophyseal origin, is in all probability due to hyperplasia or supersecretion of the pars intermedia.

On account of its direct action on unstriped muscle, extracts of the hypophysis should be especially useful in the treatment of uterine hemorrhage, and it has been used to induce labor. It has been generally taught that the pressor substance is only present in the posterior lobe. We (Lewis, Matthews, Miller) have been able to demonstrate conclusively that it is a secretion of the pars intermedia. As the anterior lobe adjacent to the cleft is histologically and embryologically pars intermedia, it should contain the pressor substance. We have shown that this is true by first removing the depressor substance present by extracting with alcohol. This pressor substance had been overlooked by previous investigators on account of the relatively small amount present in the anterior lobe.

There has been considerable speculation in regard to the manner in which the pressor substance reaches the general circulation. Herring advanced the theory that certain hyalin bodies originating in the pars internedia and detected in the pars nervosa, represent this pressor substance on its way to the stalk through which it passes to the third ventricle. Cushing modified this theory by suggesting that these hyalin bodies originating in the pars intermedia were activiated or acquired these pressor characteristics in the pars nervosa. Since we have been able to demonstrate that an active pressor substance is present in the pars intermedia, Cushing's modification becomes untenable. While hyalin bodies have been demonstrated wandering through the pars nervosa and stalk to the third ventricle, we have no conclusive evidence that they represent the pressor substance.

In addition to this pressor agent, all portions of the hypophysis contain a depressor substance. Some believe this is specific in character; others that it is the depressor substance normally found in all tissues. We have made an observation which requires confirmation, that a solution containing the depressor substance from the brain when heated in an autoclave no longer lowers blood pressure, while the same treatment did not destroy the depressor substance obtained from the hypophysis.

### CORRELATION BETWEEN THE HYPOPHYSIS AND OTHER DUCTLESS GLANDS.

The correlation of organs is one of the most interesting and complicated problems confronting the physiologist at the present time. That peculiar influence protective in character, emanating from an organ, to modify the secretion of some distant gland, verges on the uncanny, and impresses us with the harmony of the organism. There is, no doubt, an inter-relation between the hypophysis and the reproductive organs. During pregnancy the anterior lobe of the hypophysis undergoes marked hyperplasia. A new type of cell is found in such abundance that a diagnosis of pregnancy can be made from this alone. We might, therefore, infer that the hypophysis is in some manner concerned in the development of the foetus. These changes in the hypophysis are probably not due to the pregnancy per se, but to the arrested functioning of the ovary during this period. Furthermore, it is well known that the mother during pregnancy becomes generally plumper, the fingers, lips and nose thicken, and in many respects take on those changes characteristic of early acromegaly. Castration of either sex may produce somewhat similar changes in the hypophysis. In women deposits of fat and more especially changes in the facial expression follow early castration, and at autopsy hyperplasia of the anterior lobe can be demonstrated. Eunuchs show this same hyperplasia of the anterior lobe and many of these individuals have exceedingly long legs and arms, and frequently attain unusual height. Castrated children undergo unusual development when they reach puberty. It is also generally conceded that girls who reach puberty late average taller than those coming to this state early, the appearance of the sexual function stopping the hyperplasia of the hypophysis and thus interfering with the individual growth. Peoples of northern climes where puberty appears later are on an average taller than those of warm climates where puberty appears earlier. It is possible that the ungainly boy of fifteen or sixteen years, with his large feet and hands and rather heavy lower jaw, owes his rather unsightly condition to hyperplasia of the anterior lobe of the hypophysis.

### HYPOPHYSIS AND THYROID.

Quite as definite a relation exists between the hypophysis and thyroid. The anterior lobe of the hypophysis undergoes marked hyperplasia after removal or degeneration of the thyroid. Removal of the thyroid in pregnant animals is followed by hyperplasia of the anterior lobe in the foetus. In hyperthyroidism the anterior lobe of the hypophysis is often atrophic, while in myxedema, the anterior lobe is hyperplastic. After removal of the hypophysis, the thyroid frequently undergoes hyperplasia.

### HYPOPHYSLS AND SUPRARENAL.

The correlation between the hypophysis and suprarenal is almost as striking. After removal of the suprarenals it is reported that the anterior lobe of the hypophysis undergoes hypertrophy and not uncommonly in acromegaly the suprarenals are much enlarged; in one instance reported by Fischer, five times the normal size.

Summarizing briefly our present knowledge of the physiology of the hypophysis, it may be said that the hypophysis is essential to life. Acromegaly is associated with hyperplasia and probably supersecretion of the glandular elements in the anterior lobe. Obesity and failure of sexual development, known as Fröhlich's syndrome is apparently due to lessened function of the posterior lobe, whether pars intermedia or pars nervosa has not been determined. The pars intermedia secretes a presser substance which has also a diuretic action. Polyuria and glycosuria, often observed in acromegaly and after experimental disturbances of the hypophysis are due probably to supersecretion of the pars intermedia. There is a correlation between the hypophysis, thyroid, suprarenals, ovaries and testes.

